# Synergistic Inhibition of Synbiotic Cultures among Lactobacilli and Plant Extracts against Vaginal Discharge Causing *Candida albicans*

**DOI:** 10.3390/nu16091372

**Published:** 2024-04-30

**Authors:** Siriwoot Sookkhee, Phadungkiat Khamnoi, Thanapat Sastraruji, Sathian Boonkum, Nitwara Wikan, Wutigri Nimlamool

**Affiliations:** 1 Department of Microbiology, Faculty of Medicine, Chiang Mai University, Chiang Mai 50200, Thailand; siriwoot.s@cmu.ac.th; 2 Diagnostic Laboratory Unit, Maharaj Nakorn Chiang Mai Hospital, Faculty of Medicine, Chiang Mai University, Chiang Mai 50200, Thailand; phadungkiat.k@cmu.ac.th; 3 Dental Research Center, Faculty of Dentistry, Chiang Mai University, Chiang Mai 50200, Thailand; s_thanapat@hotmail.com; 4 Department of Biotechnology, Faculty of Agro-Industry, Chiang Mai University, Chiang Mai 50200, Thailand; sathian.b@cmu.ac.th; 5 Department of Pharmacology, Faculty of Medicine, Chiang Mai University, Chiang Mai 50200, Thailand; nitwara.wik@cmu.ac.th; 6 Lanna Rice Research Center, Chiang Mai University, Chiang Mai 50200, Thailand

**Keywords:** *Candida albicans*, plant extract, synbiotic, *Lactobacillus crispatus*, *L. reuteri*, *H. tuberosus*

## Abstract

Vulvovaginal candidiasis (VVC) is the most common cause of vaginal discharge among women. The present study aimed to investigate the synergistic anticandidal effect of lactobacillus cultures supplemented with plant extracts. Among 600 isolates of lactic acid bacteria, 41 isolates exhibited inhibitory activity against *Candida albicans* ATCC10231. Six out of 41 cell-free supernatants demonstrated the most potent antibacterial and anticandidal activities. They also inhibited the clinical isolates of *C. albicans,* causing VVC and non-*C. albicans*. The synergistic effect between *Lactobacillus crispatus* 84/7 and *Limosilactobacillus reuteri* 89/4 was demonstrated by the lowest fractional inhibitory concentration index (FICI = 0.5). The synbiotic culture of bacterial combination, cultured with Jerusalem artichoke (*H. tuberosus*) extract, also exhibited the strongest inhibition against the tested *C. albicans*. Biofilm formation decreased after 12 h of incubation in the selected cell-free supernatants of this synbiotic culture. The anticandidal activity of crude extracts was lost after treatment with proteinase K and trypsin but not with heating conditions, suggesting that it may be a heat-stable substance. In conclusion, the combination of *L. crispatus* 84/7 and *L. reuteri* 89/4 with *H. tuberosus* may be a promising candidate for inhibiting *Candida* infection and biofilm formation, with the potential use as ingredients in vaginal biotherapeutic products.

## 1. Introduction

*Candida* spp. is one of the most common causes of candidiasis, with *C. albicans* being the most important one [1]. Currently, approximately 75% of women experience vulvovaginal candidiasis (VVC) one or more times [2]. The development of resistant fungi and therapy failures following long-term use of antifungal drugs has increased, particularly in immunocompromised patients [3]. Due to the inappropriate usage of antifungal agents leading to drug resistance in vaginal *Candida*, there remains a need for alternative anticandidal agents to control pathogenic *Candida* infections [4]. Therefore, the search for alternatives, such as bacteriocin, which could better control and treat microbial infections, has been suggested [5]. As part of the vaginal microflora, lactobacilli can help prevent vulvovaginal infections [6]. The antifungal activities of lactobacilli are not sorely attributed to hydrogen peroxide, a typical antimicrobial factor in lactobacilli [7]. The potent antifungal activity of *Lactobacillus* can also be partially attributed to the low pH and the production of organic acids [8]. Additionally, lactobacilli can produce antimicrobial peptides, such as bacteriocins [9]. Bacteriocins are low-molecular-mass peptides synthesized by bacterial ribosomes and released extracellularly to kill or inhibit other pathogenic bacteria [10]. Moreover, aside from bacteria, bacteriocin-like inhibitory substances are substances with bacteriocin-like properties that can inhibit other microorganisms, including *Candida* [11]. The interest in applying bacteriocins in food preservation has grown in recent years due to their non-toxicity, sensitivity to proteases, general pH and heat stability, and antimicrobial effects against both Gram-positive and Gram-negative bacteria [12,13,14]. Recently, we discovered a bacteriocin harvested from the synbiotic culture of lactobacilli with various plant extracts, especially *Lactocaseibacillus paracasei* with nashi pear extract [15]. But, no investigations have focused on the synergistic anticandidal effect of lactobacillus culture with plant extract as the prebiotic-enriched source. The fungicidal effect of plantaricins (Pln E/F and J/K) against *C. albicans* was investigated by Sharma and Srivastava [16]. Additionally, Santos et al. [17] demonstrated that bacteriocins produced by *Lactobacillus plantarum* have an anti-inflammatory effect against *C. albicans*, while Graham et al. showed that bacteriocin EntV from *Enterococcus faecalis* has potential as a novel antifungal agent against *C. albicans* [18]. Several investigators have demonstrated the anticandidal effects of different *Lactobacillus* species [8,16,19,20,21,22]. However, there is a lack of reports investigating the anticandidal activity and/or synergistic anticandidal activity against vaginal candida that causes vulvovaginal candidiasis. The aim of this study was to investigate the anticandidal activity of *Lactobacillus* and to evaluate the enhancement of prebiotic in the tested plant extracts for synbiotic culture with probiotic lactobacilli, with the ultimate goal of applying it therapeutically against this pathogenic yeast.

## 2. Materials and Methods

### 2.1. Lactic Acid Bacteria

Six hundred isolates of LAB, which were formerly recovered from raw milk, fermented milk, yogurt, cheese, meat products, mixed pickles, and clinical specimens of humans in a previous study reported by our colleagues from 2006 to 2021, were used. A purified colony of each LAB was isolated in de Mann–Rogosa–Sharpe broth (MRS; Bacto^TM^; Bacton Dickinson, Sparks, MD, USA) under a 5% CO_2_ atmosphere at 37 °C for 48 h before being transferred into an aliquot tube containing glycerol as a cryoprotectant. They were then stored in a deep freezer managed by Dr. Siriwoot Sookkhee, Department of Microbiology, Faculty of Medicine, Chiang Mai University, until use.

To identify these isolates, each one was confirmed to the species level using API-50 CHL biochemical identification [15,23] or the VITEK-MS apparatus [24]. Briefly, two drops of a McFarland Standard No. 2-adjusted LAB suspension in MRS broth were inoculated into API-50 CHL medium (bioMerieux^TM^, Marcy l’ Etoile, France) before being covered with a drop of sterilized mineral oil. Based on the fermentation profiles, the species of each isolate was identified after 24 and 48 h of incubation using the API^®^50 CHB/E databases. To identify the species by analyzing the proteomic profiles, a single colony of each isolate was smeared onto a well of VITEK MS-DS target slides (bioMerieux^TM^, Marcy l’ Etoile, France). Alpha-4-cyano-4-hydroxycinnamic acid (CHCA) matrix (bioMerieux^TM^, Marcy l’ Etoile, France) was then applied to each well to facilitate crystallization. The VITEK-MS apparatus was run according to the conditions described in a previous report [25]. Peptide spectra from each well of the target slide were determined and then processed to interpret the species identification using the Myla^®^ database (bioMérieux^TM^, Marcy l’ Etoile, France). The numbers and species of these LABs are shown in Table 1.

### 2.2. Microbial Indicators

*C. albicans* ATCC10231 was used as the indicator in the agar-spot assay to screen for anticandidal activity. To investigate the anticandidal activity, twelve clinical isolates of *C. albicans* and non-*C. albicans* were collected from SDA plates of vulvovaginal candidiasis (VVC) specimens. These specimens were kindly provided by the Diagnostic Laboratory Section, Maharaj Nakorn Chiang Mai Hospital, Faculty of Medicine, Chiang Mai University, Thailand, in 2019. *Candida* isolates were separately cultured on Sabouraud Dextrose agar (SDA; Difco^TM^, Becton Dickinson, Sparks, MD, USA) and incubated at 37 °C for 24 h. Isolated colonies were identified using colony characteristics, Gram staining, and the germ tube test before being confirmed using VITEK-MS for molds and yeasts (BioMèrieux, Marcy l’ Etoile, France).

*Staphylococcus aureus* ATCC 25923, *Sarcina lutea* ATCC 9341, *Escherichia coli* ATCC 25922, and *Bacillus subtilis* ATCC 6633 were used as tested indicators to investigate the antimicrobial activity of probiotics using agar-cup diffusion assay [26]. They were also stored in a deep freezer in the Bacteria Unit of the Department of Microbiology, Faculty of Medicine, Chiang Mai University, Thailand. Bacterial indicators were separately propagated in Tryptic Soy broth (TSB; Difco^TM^, Becton Dickinson, Sparks, MD, USA). *Candidal* isolates were grown in Sabouraud Dextrose broth (SDB; Difco^TM^, Becton Dickinson, Sparks, MD, USA) at 37 °C for 24 h before being adjusted to a turbidity equivalent to McFarland No. 0.5.

### 2.3. Screening of Anticandidal Activity

The anticandidal screening of the LAB isolates was performed by an agar-spot assay as described in the previous study [19]. *C. albicans* ATCC10231 was used as the tested indicator. Each LAB isolate was grown on de Mann–Rogosa–Sharpe (MRS; Difco^TM^, Becton Dickinson, Sparks, MD, USA) agar and then incubated under CO_2_ condition for 24 h. This LAB culture agar was then overlaid with the molten SDA plate that was supplemented with *C. albicans* ATCC10231 previously grown at 37 °C for 24 h. After the overlaid agar was set, this plate was incubated at 37 °C for 24 h. A colony of LABs that gave the inhibition zone around its colony was selected for further investigation.

### 2.4. Antibacterial Activity of the Selected Anticandidal Probiotic Isolate

The antibacterial activity was determined by using an agar-well diffusion method against the reference strains of *S. aureus, S. lutea*, *B. subtilis,* and *E. coli,* as described in the previous report [26]. Each overnight culture, adjusted to a turbidity equal to McFarland Standard No. 0.5, was separately swabbed on Mueller Hinton Agar (MHA; Bacto^TM^, Becton Dickinson, Frankin Lake, NJ, USA) plates, and wells were made using a cork borer. The cell-free supernatant of each LAB, which was cultured in MRS broth under CO_2_ condition for 48-h cultivation, was filled into the wells on the swabbed agar before incubating at 37 °C for 24 h. The experiments were conducted in duplicate. Antimicrobial activities against these tested indicators were evaluated based on the average inhibition zones.

### 2.5. Determination of Anticandidal Activity

The antimicrobial activity of these selected isolates against 12 clinically tested *Candida* strains was investigated using an agar-cup diffusion assay [27]. The cell-free supernatant of selected LAB was freshly prepared and filled into the cylinder cup, which was then placed on the swabbed SDA plates and incubated at 37 °C for 24 h. The inhibitory activities against these tested isolates were evaluated based on the average inhibition zones. The experiments were conducted in triplicate. LAB isolates that exhibited the largest inhibition zones were selected for further studies as potent anticandidal isolates.

### 2.6. Preparations of Plant Powder Extracts

Twenty-eight plants, as shown in Table 2, including 8 cereals, 5 vegetables, 9 fruits, 2 medicinal plants, and 4 tuber plants, were prepared using a soybean machine, juice extractor, or food mixer. Their powders were produced using the freeze-dried technique (CHRIST ALPHA1-4, Memmingen, Germany). Inulin (Merck™; Merck KGaA, Darmstadt, Germany) at a concentration of 2% *w*/*v* was used as the positive control. These plants were organically grown and provided by The Organic Plantation of Royal Project Foundation, Chiang Mai, Thailand. These plant extracts were required so as not to inhibit the growth of the synbiotic culture of LAB [15].

### 2.7. Preparation of the Cell-Free Supernatant of Synbiotic Cultures

Synbiotic cultures were performed as described in our previous study [15]. Briefly, each 5% *w*/*v* plant powder suspension in the modified MRS broth (PM) was sterilized by autoclaving at 110 °C, 15 pounds for 10 min. After 48 h of LAB culture in MRS broth, the culture was centrifuged at 4121× *g* at 4 °C. The pellet was resuspended in Phosphate Buffer Solution (PBS) pH 7.2 to obtain an initial LAB concentration equivalent to McFarland No. 2. The modified MRS broth (M), with the carbon source removed, was used as the synbiotic culture media. Each selected LAB strain was separately cultured in the presence (synbiotic culture or LPM) and absence (probiotic culture or LM) of 5% *w*/*v* plant powder extract in the modified MRS broth, with a volume ratio of L:P:M at 2:3:5. After 48 h of incubation, cell-free supernatant was harvested after being centrifuged at 4121× *g* for 10 min.

### 2.8. Determination of Minimal Inhibitory Concentration (MIC)

The anticandidal activities against *C. albicans* ATCC10231 of each isolate among these systems were determined using the broth microdilution assay [26]. Synbiotic cultures that exhibited the lowest MIC values against the clinically tested strains were selected for further study. Briefly, the stock solution of each extract was 2-fold serially diluted with SDB to a total of 10 concentrations in 96-well microtiter plates (Corning™, Corning Inc., Salt Lake City, UT, USA). The highest final concentration of the tested supernatant was 512 µg/mL. Each purified colony of the tested isolate was inoculated into SDB, and the candida suspension was adjusted to 0.5 McFarland standard turbidity [15,28]. After a 24-h incubation, each well was observed optically for either growth or complete inhibition of growth. MIC values were then recorded. Each tested compound was evaluated in triplicate. The cell-free supernatants of the isolates that provided the lowest MIC value against the clinically tested strains were selected for further assays, and this was used as a screening criterion for potential probiotics.

### 2.9. Fractional Inhibitory Concentration Index of Anticandidal Activity of LAB Culture Combination

The standard checkerboard assays were performed as described previously to evaluate the combination effect of each tested lactic acid bacteria and the plant extracts against *C. albicans* ATCC10231 [29]. Fifty microliters of LM supernatant dissolved in MRS broth was serially diluted with MRS broth along the vertical axis of rows 1st–7th (top and bottom: the highest and lowest final concentrations were performed in titers at 1 and 7, respectively). In the 8th row, the 2-fold serially diluted supernatant was not put into the wells; therefore, this row showed only the action of PM or was referred to as “PM alone”. Fifty microliters of the serial two-fold dilutions of PM in MRS broth was added to each well along the horizontal axis of the 96-well microtiter plates in columns 1st–7th (left and right: the highest and lowest final concentrations were performed in titers at 1 and 7, respectively). In the 8th right column of the 96-well plate, the 2-fold serially diluted PM was not put into the wells, and this column showed only the action of LM or was referred to as “LM alone”. In the 9th right column of the 96-well plate, each well was filled with 50 µL of SDB and 50 µL of MRS as the negative control or referred to as an “untreated well”. These tested isolates were confirmed for susceptibility by determining MIC values for chlorhexidine. In the 10th right column of the 96-well plate, each well was filled with 50 µL of chlorhexidine solution and 50 µL of MHB as the positive control. From an overnight culture, a suspension was prepared in SDB broth that corresponded to 0.5 McFarland turbidity. Then, 0.1 mL of this suspension was dissolved in 9.9 mL of MHB, resulting in an inoculum containing approximately 5 × 10^5^ CFU/mL at the final bacterial concentration. Afterward, 100 µL of the inoculum was added to each well of the 96-well microdilution plates. The inoculated microplates were incubated at 37 °C for 24 h. After 24 h, MIC was defined as the lowest concentration that inhibited the growth of the test microorganism [30]. All experiments were performed in triplicate. The Fractional Inhibitory Concentration Index (FICI) value was used to assess whether synergism, indifference, or antagonism occurred following the combination effects of LM and PM employing the following formula [29]:

FIC_LM_ = MIC_LM in combination_/MIC_LM_

FIC_PM_ = MIC_PM in combination/_MIC_PM_

FICI = FIC_LM_ + FIC_PM_

The combination effect was evaluated based on the following criteria:

FICI ≤ 0.50 denoting synergism;
0.50 <FICI ≤ 0.75 denoting partial synergy;
0.75 < FICI ≤ 1 denoting an additive effect;
1 < FICI ≤ 4 denoting indifference or no interaction;
and FICI > 4 denoting antagonism.

### 2.10. Biofilm Preparation

One hundred microliters of *C. albicans* ATCC10231, adjusted to an optical density of McFarland No. 1, was dropped to each well of the pellicle-prepared 96-well plate. Fifty microliters of SDB was separately added to each well. Fifty microliters of cell-free vaginal discharge was also separately added to each well. The tested 96-well plate was then incubated at 37 °C for 6 h for initial adherence. After the incubation period ended, the entire supernatant in each well was gradually decanted before being washed twice with 200 μL of PBS solution. One hundred microliters of SDB was immediately added to each well and replaced every 24 h until the incubation was completed after 48 h. After the incubation period ended, the entire supernatant in each well was completely drained before being washed twice with 200 μL of PBS solution. The growth of candidal biofilm was then detected.

### 2.11. Inhibition of Candidal Viability and Biofilm Formation Detected by Fluorescence Microscopy

The freshly prepared biofilm of *C. albicans* was assessed at 48 h of the incubation period, as described in the previous report [31]. *C. albicans* biofilms formed on the surface of the coverslip were then incubated with the selected synbiotic culture of potent LAB and *H. tuberosus* for 12 h. The coverslip was washed twice with PBS, and then the solution was removed before staining with FUN^TM^-1 to determine candida viability and the appearance of the biofilm extracellular matrix. Prior to staining, a coverslip was transferred to a new well in a 6-well plate and incubated with 2 mL of PBS containing 10 µM FUN^TM^-1 fluorescent dye (Invitrogen, Thermo Fisher Scientific, Waltham, MA, USA) and 25 µg/mL of concanavalin A (Con A)-Alexa Fluor 488 conjugate (Invitrogen, Thermo Fisher Scientific, Waltham, MA, USA) for 45 min at 37 °C in a dark condition [32]. This investigation was conducted in triplicate. After the incubation period, fluorescence microscopy (Olympus EX41 microscope, Olympus Corp., Shenzhen, China) was performed to observe the candidal viability at 400× magnification.

### 2.12. Characterization of Inhibitory Substances

The cell-free supernatants of the selected LAB isolates in each LPM and LM culture were further characterized. The residual anticandidal activity against *C. albicans* ATCC10231 under different conditions was determined and compared with the positive control (untreated, 100% activity), as described in our previous report [26]. For pH sensitivity, each supernatant was adjusted to pH 3.0, 5.0, 7.0, and 10.0 using hydrochloric acid (HCl) and sodium hydroxide (1 mol/L NaOH) (Sigma-Aldrich, St. Louis, MO, USA), and then incubated at 37 °C for 15 min. For heat treatment, each supernatant was separately incubated in a water bath at 60 °C, 80 °C, 100 °C for 30 min, and 121 °C for 15 min. For hydrogen peroxide production, each supernatant was treated with a 1 mg/mL solution of catalase (Sigma-Aldrich, St. Louis, MO, USA) and then incubated at 37 °C for 15 min. For proteolytic enzyme sensitivity, each bacteriocin was separately treated with the final concentration of 1 mg/mL of protease, trypsin, and pepsin. Following incubation at 37 °C for 30 min, enzyme activity was terminated by adding the excess fetal bovine serum, and the remaining activities were determined.

### 2.13. One-Dimensional Polyacrylamide Electrophoresis

The selected anticandidal bacteriocins were analyzed using glycine or tricine sodium dodecyl sulfate-polyacrylamide gel (Mini-Protein™ IV, BIO-RAD laboratory, Hercule, CA, USA) with a multicolor low-range protein ladder (Spectra™, Fermentus, Vilnius, Lithuania). They were visualized using the PlusOne Silver Staining Kit (PlusOne™, GE HEALTHCARE (AB)”, Pollards Wood, Nightingales Lane, Chalfont St. Giles, Buckinghamshire, HP8 4SP, UK). The molecular weights of the protein bands were calculated.

### 2.14. Partial Purification of Potent Bacteriocins

Overnight cultures of the potential probiotic isolates propagated in the LPM culture system were adjusted to be equal to McFarland No. 2. Cultures were centrifuged at 12,000× *g* for 15 min at 4 °C, and the obtaining supernatant was designated as a cell-free supernatant. To neutralize hydrogen peroxide, 1 mg/mL of bovine catalase was added. The pH of each cell-free supernatant was adjusted to 6.5 with 1 mol/L NaOH. The treated solution was designated as bacteriocin-like substances. Bacteriocins from these potent supernatants were partially purified. Using vivaflow-50™ ultrafiltration, the cell-free supernatant of each selected LAB isolate was fractionated with cut-offs at 10 and 30 kDa (Sartorius Stedem Lab, Stonehouse, UK), harvested, and then concentrated. Each bacteriocin was separately purified by anion exchange chromatography using Fast Protein Liquid Chromatography (FPLC; AKTA^®^ Explorer, GE Healthcare Life Science, Marlborough, MA, USA). Hitrap^TM^ Capto S, a strong sulfoethyl cation exchanger (GE HEALTHCARE (AB)”, Pollards Wood, Nightingales Lane, Chalfont St. Giles, Buckinghamshire, HP8 4SP, UK), was performed as a stationary phase. Bacteriocin fractions after elution with 20 mM Tris-HCl pH 8.0 were separately collected and desalted. Their concentrations were determined by Qubit fluorometric assay. Anticandidal activity against *C. albicans* ATCC10231 was determined by agar-well diffusion assay.

### 2.15. Statistical Analysis

A scattered dot plot of the median value with an interquartile range and bar of mean value with standard deviation value was created using GraphPad Prism version 9.0.0 for Windows, GraphPad Software, San Diego, CA, USA. One-way ANOVA and Tukey’s multiple comparisons at *p* < 0.01 were performed as the statistical analysis in the present study by using the software IBM SPSS Statistics (Statistical Package for the Social Sciences) for Windows Version 25 (IBM Corp., Armonk, NY, USA).

## 3. Results

### 3.1. Screening of Anticandidal Activity

Interestingly, among the 600 tested isolates of LAB, 41 demonstrated anticandidal activities against *Candida albicans* ATCC10231 using agar-spot assay as the screening step. Ninety isolates of thirteen species were not selected as the anticandidal isolates, namely, *B. lactis* (*n* = 6), *B. longum* (*n* = 12), *C. kimchi* (*n* = 1), *F. fructivorans* (*n* = 1), *L. casei* (*n* = 24), *L. johnsonii* (*n* = 15), *L. helveticus* (*n* = 21), *L. kefiri* (*n* = 1), *L. cerevisiae* (*n* = 1), *L. capillatus* (*n* = 3), *L. brevis* (*n* = 3), *S. kimchicus* (*n* = 1), and *S. oryzae* (*n* = 1). Among 106 isolates of five species, only one isolate of each species, namely, *L. acetotolerans*, *L. acidophilus*, *L. delbrueckii* subsp. *delbrueckii*, *L. jensenii*, and *L. lactis* were selected after exhibiting an inhibition zone around the colony. Apart from the 404 isolates of 10 species, only 36 isolates with anticandidal properties were selected, including 2 of *L. sakei* subsp. *sakei*, 2 of *L. buchneri*, 2 of *P. acidilactici*, 3 of *L. curvatus*, 4 of *L. fermentum*, 4 of *L. paracasei* subsp. *paracasei*, 4 of *L. plantarum* subsp. *plantarum*, 4 of *L. rhamnosus*, 5 of *L. reuteri*, and 6 of *L. crispatus.* These isolates possessed potent anticandidal properties and were selected.

After the secondary screening by agar cup diffusion assay (where the lowest MIC value indicated the best activity), only six out of 41 cell-free supernatants exhibited the most potent antibacterial activities against the standard bacterial strains: *S. aureus* ATCC 25923; *S. lutea* ATCC 9341; *E. coli* ATCC 25922; and *B. subtilis* ATCC 6633. The inhibition zones of these isolates are shown in Table 3. They possessed both anticandidal and antibacterial activities and were selected. These selected isolates were *Lacticaseibacillus rhamnosus* 68/7, *Latilactobacillus curvatus* 87/6, *Limosilactobacillus fermentum* 20/6, *Lactobacillus crispatus* 84/7, *Lacticaseibacillus paracasei* subsp. *paracasei* 9/5, and *Limosilactobacillus reuteri* 89/4.

**Figure 1:**
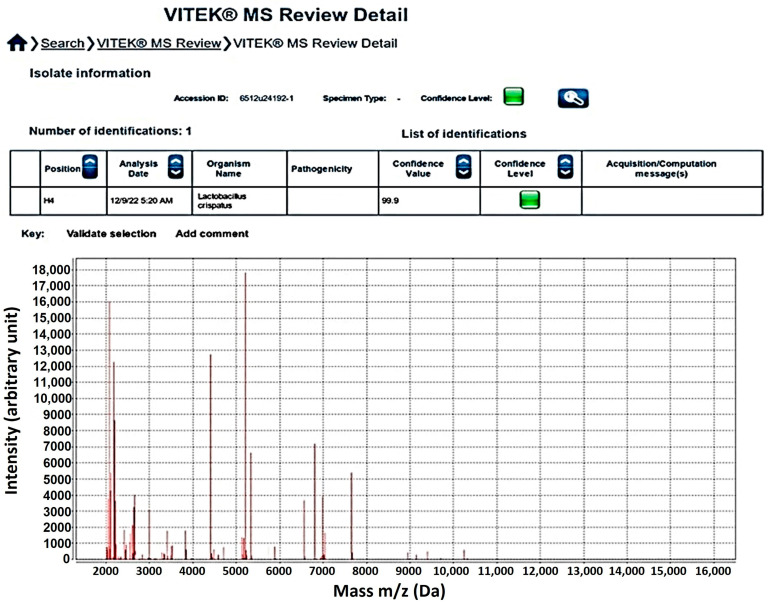
Identification result of *L. crispatus* 84/7 after detected by Vitek MS and Myla database.

The species identification was acceptable at the level of 80% identity. Six selected isolates could be identified by using API^®^50 CHB/E and Myla^®^ database; for example, the identification of *L. crispatus* 84/7 according to Vitek MS and Myla database is shown in Figure 1.

### 3.2. Determination of Anticandidal Activity

**Figure 2:**
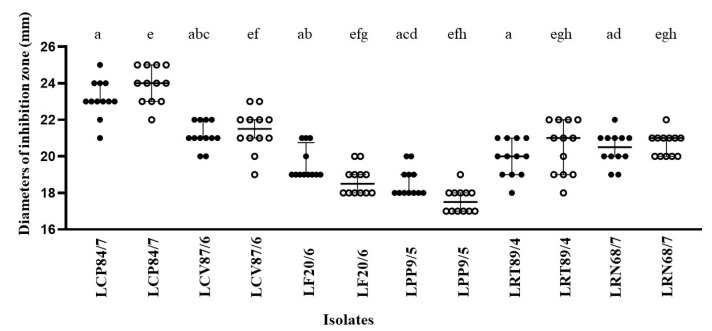
Anticandidal activities of these isolates after tested using agar-cup diffusion (●, *C. albicans* (*n* = 12); ◯, non-*C. albicans* (*n* = 12) isolates). Significant differences shown as the alphabet; *p* < 0.001 (for a, c–h), and *p* = 0.004 (for b).

**Figure 3:**
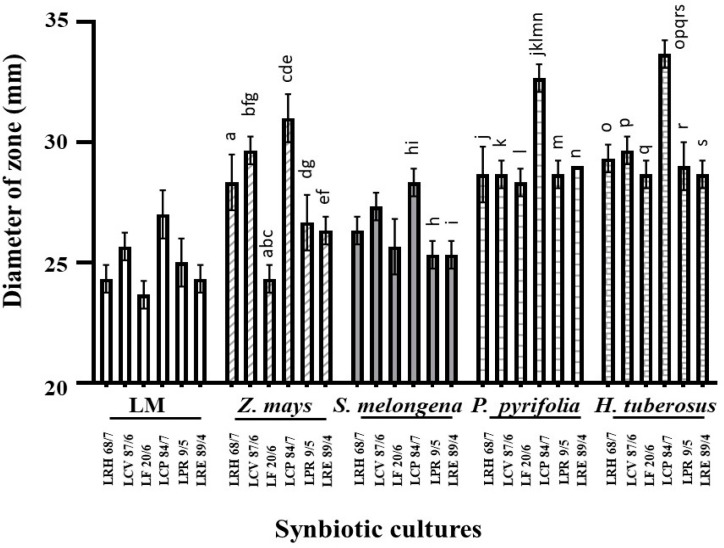
Anticandidal activity of the cell-free supernatant of synbiotic cultures determined by the diameter of inhibition zone. Data are shown as the average diameter of inhibition zone (mm). a–s represent the significant difference at *p* < 0.001, except g is at *p* = 0.002.

The anticandidal activities of these isolates are shown in Figure 2. These isolates were identified as potent anticandidal isolates. However, no significant difference in the activity was observed when comparing the effects on *C. albicans* and non-*C. albicans*. In Figure 3, *L. crispatus* 84/7 demonstrated the largest median values of the diameter of the inhibition zone against both VVC-causing *C. albicans* (*n* = 12) and non-*C. albicans* (*n* = 12) and exhibited significant differences compared to all other isolates (a and e).

### 3.3. Anticandidal Activity of the Cell-Free Supernatant of Synbiotic Cultures

The LPM synbiotic cultures of the selected LAB isolates and plant extracts were evaluated based on the growth of LAB isolates. The growth enhancement of these LPM synbiotic cultures is demonstrated in Table 4. The results showed that the biggest fold change in LPM growth occurred with selected isolates when using cereal, vegetable, fruit, and tuber plant extracts, including *Z. mays* (No. 6), *S. melongena* var. *serpentinum* (No. 11), *P. pyrifolia* (No. 19), *H. tuberosus* (No. 25), respectively. It is suspected that these selected plant extracts contain prebiotic compounds that enhance the growth of these isolates in LPM compared to their growth in LM. Due to the prebiotic effect, these tested plants may be important sources that synergistically affect the antimicrobial activity of these selected antimicrobial LAB isolates, as shown in Table 4. These included *Z. mays* (No. 6), *S. melongena* var. *serpentinum* (No. 11), *P. pyrifolia* (No. 19), and *H. tuberosus* (No. 25). The potent activity was demonstrated in the LPM cultures of these plant extracts when compared to their inhibitions in LM cultures. These plant extracts were selected as the prebiotic source for the synbiotic culture of these isolates against *C. albicans* ATCC10231.

**Figure 4:**
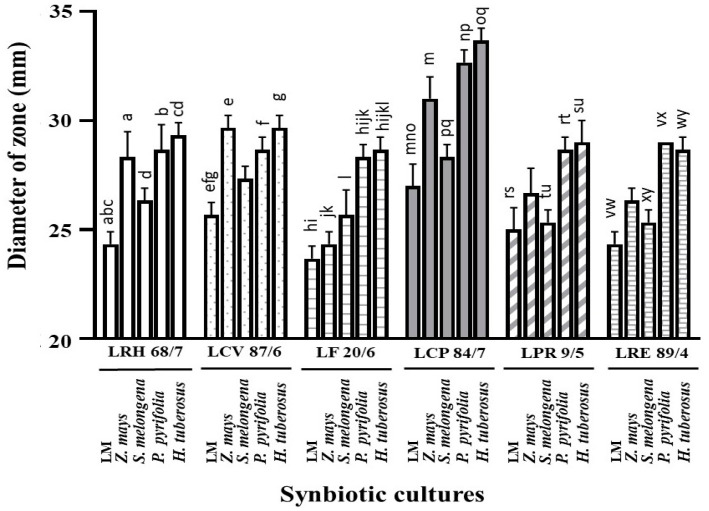
Anticandidal activity of synbiotic cultures between each plant powder extract with each isolate. Data are shown as the average diameter of inhibition zone (mm). a–y represent the significant difference at *p* < 0.001, except g is at *p* = 0.002.

From the results of each selected LAB isolate, it was found that the larger inhibition zones were demonstrated in the LPM synbiotic culture compared to bacterial cultures alone (LM). It was noted that these plant extracts can act as natural prebiotics, supporting the increased growth and anticandidal activity of such isolates. Among the six LPM cultures of these isolates, the cultures with the powder extracts of *P. pyrifolia* (No. 19), *H. tuberosus* (No. 25), and *Z. mays* (No. 6) showed strong anticandidal activity when determining the average inhibition zone against the tested strains of *C. albicans*. LPM synbiotic cultures of *L. curvatus* 87/6, *L. crispatus* 41/9, and *L. reuteri* 89/4 with *P. pyrifolia* or Nashi pear (No. 19) also exhibited the highest activity in the same manners (Figure 3 and Figure 4). It was a prebiotic-rich fruit that supported the growth and antimicrobial activity of these LAB isolates. Besides the prebiotic source of fruit, *Z. mays* or sweet corn (No. 6), which was the only selected cereal, showed potent antimicrobial activity in the LPM synbiotic culture of this isolate. Lastly, *H. tuberosus* or Jerusalem artichoke (No. 25), well-known as a high-rich inulin source, was also selected for its potent antimicrobial activity.

### 3.4. Fractional Inhibitory Concentration Index of Anticandidal Activity of LAB Culture Combination

**Figure 5:**
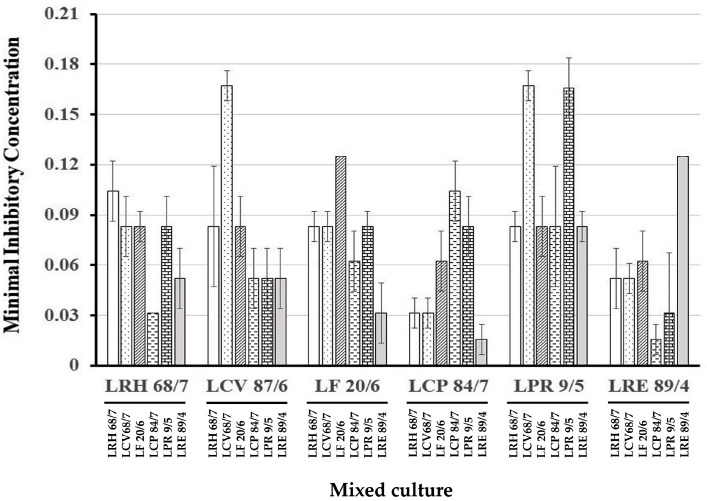
Minimal inhibitory concentration (MIC) of each culture combination of LAB. Initial concentration of each cell-free supernatant is assumed to be equal to 1.0. The two-fold dilution was performed, and the concentration was obtained.

**Figure 6:**
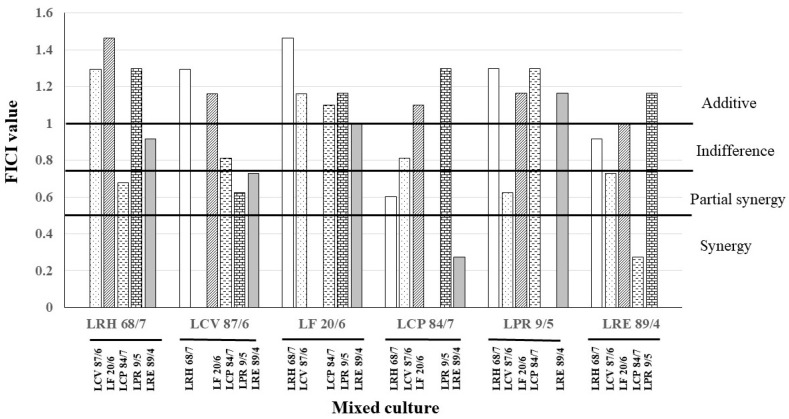
FICI values of each culture combination of LAB. The synergistic, partial synergistic, indifference, and additive effects are interpreted according to FICI value against *C. albicans* ATCC 13803.

Based on the anticandidal activity of the six selected LAB isolates, their minimal inhibitory concentrations against *C. albicans* ATCC10231 were determined. The results revealed that some culture combinations of LAB isolates demonstrated lower MIC concentrations than the cultures of LAB isolate alone, as shown in Figure 5. The FICI value of each combination was calculated and is displayed in Figure 6. The synergistic effect was observed only in the culture combination of *L. crispatus* 84/7 with *L. reuteri* 89/4 (FICI = 0.28) and *L. reuteri* 89/4 with *L. crispatus* 84/7 (FICI = 0.28). Six combinations showed partial synergy, namely, the culture combination between *L. rhamnosus* 68/7 with *L. crispatus* 84/7 (FICI = 0.60), *L. curvatus* 87/6 with *L. paracasei 9/5* (FICI = 0.62), *L. curvatus* 87/6 with *L. reuteri* 89/4 (FICI = 0.73), *L. crispatus* 84/7 with *L. rhamnosus* 68/7 (FICI = 0.68), *L. paracasei* 9/5 with *L. curvatus* 87/6 (FICI = 0.62), and *L. reuteri* 89/4 with *L. curvatus* 87/6 (FICI = 0.73). Other combinations did not show synergistic probiotic cultures. In this study, we were particularly interested in the culture combination of *L. crispatus* 84/7 with *L. reuteri* 89/4. Consequently, this combination was selected to investigate the synbiotic cultures with three selected plant extracts. Furthermore, the selected synbiotic culture (LPM) of the probiotic combination, *L. crispatus* 84/7 with *L. reuteri* 89/4, supplemented with a powder extract of *H. tuberosus,* exhibited the enhanced anticandidal activity compared to each probiotic culture alone (LM_84/7_, and LM_89/4_), as demonstrated in Table 5.

### 3.5. Inhibition of Candidal Viability and Biofilm Formation Detected by Fluorescence Microscopy

**Figure 7:**
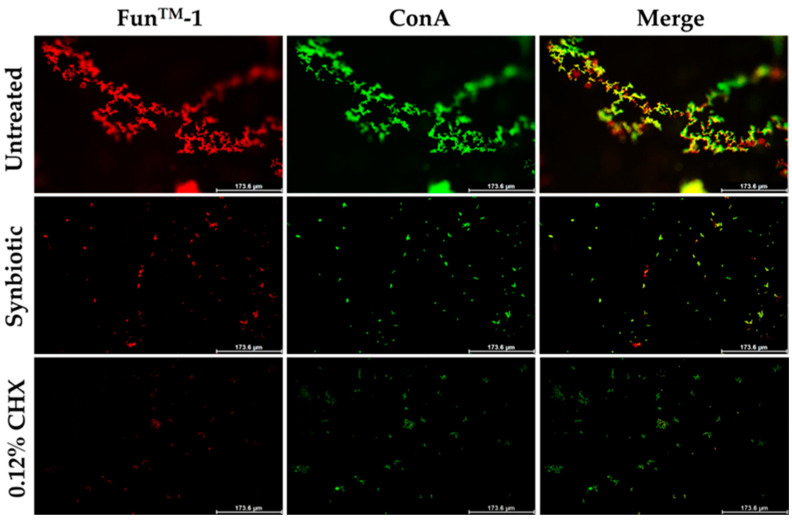
Inhibition of candidal viability and biofilm formation detected by fluorescence microscopy after 8 h of immersion. Biofilm formation of *C. albicans* ATCC10231 in SDB, Synbiotic, and 0.12% CHX solution, respectively, detected by Fun^TM^-1 (red) and ConA (green). CHX was the control.

The synbiotic culture of selected LAB isolates (the probiotic combination, *L. crispatus* 84/7 with *L. reuteri* 89/4), in combination with *H. tuberosus,* showed biofilm inhibition after a 12-h immersion. Biofilm formation of the tested *C. albicans* was stained with fluorescent dyes. The red fluorescence signal of FUN^TM^-1 appeared to be concentrated in candidal cells, indicating that the *C. albicans* cells were alive. Figure 7 reveals that biofilm formation and its extracellular matrix decreased after immersion in the selected cell-free supernatant of mixed synbiotic culture strains, compared to no treatment and a 0.12% CHX solution used as the negative and positive controls, respectively. Therefore, this synbiotic culture of potent LAB combination and *H. tuberosus* was preferentially selected for its anticandidal activity and antibiofilm effects during a 12-h immersing period.

### 3.6. Characterization of Inhibitory Substances

According to the determination of the remaining activity after treatment with various physical and chemical conditions, complete inactivation of the anticandidal activity of the selected LPM cell-free supernatant was observed after treatment with protease and trypsin, when compared to the activity of the LM cell-free supernatant. However, the anticandidal activity of the crude extracts only slightly decreased at 121 °C for 15 min (residual activity = 78%), 100 °C for 30 min (residual activity = 86%) over a wide pH range of 3, 5, 7, and 8 (residual activity = 78%, 78%, 96%, and 82%, respectively), and the addition of catalase (87%). These results suggest that they can be confirmed as heat-stable proteinaceous compounds.

### 3.7. One-Dimensional Polyacrylamide Electrophoresis

**Figure 8:**
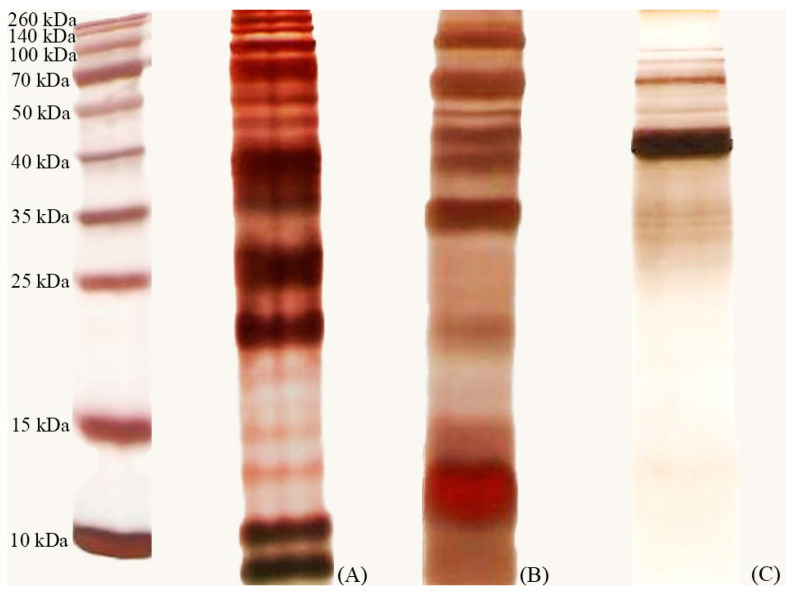
One-dimensional SDS-PAGE gel of crude protein harvested from (**A**) cell-free supernatant of synbiotic culture compared with (**B**) cell-free supernatant of *L. crispatus* 84/7 alone; (**C**) cell-free supernatant of *L. reuteri* 89/4 alone.

Figure 8 displays the protein profile of crude proteins harvested from the synbiotic culture of probiotic combination. Two areas are of particular interest: a low molecular weight region at approximately 10 kDa with two bands and a median molecular weight area ranging from about 20–30 kDa. Using vivaflow-50^TM^ ultrafiltration with cut-offs at 10 and 30 kDa, we collected two fractions within the ranges of <10 kDa and 10–30 kDa and then desalted and concentrated them. Only the fraction within the <10 kDa range exhibited anticandidal activity, prompting us to select it for further purification.

### 3.8. Purification of Potent Bacteriocins

**Figure 9:**
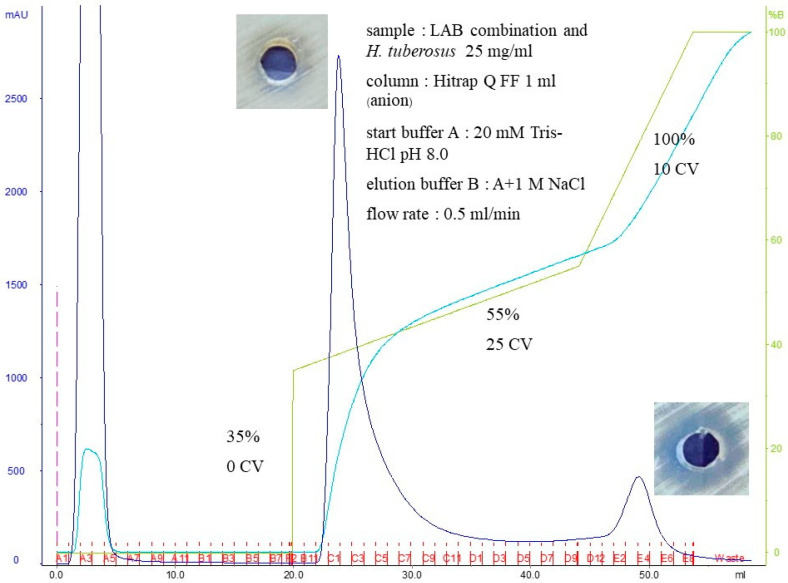
Elution of the bacteriocin harvested from the synbiotic culture of potent LAB combination and *H. tuberosus*. Anticandidal activity and SDS-PAGE are also shown. Dark blue line, UV absorbance of protein amount at 280 nm; light blue line, % salt condition; green line, salt concentration; red line, fraction number; violet line, sample injection.

Using anion exchange chromatography, the bacteriocin from the synbiotic culture of a potent LAB combination and *H. tuberosus* was eluted, as shown in Figure 9. However, the first main elution of bacteriocin did not exhibit anticandidal activity. The activity against *C. albicans* ATCC10231 was detected in the second elution. Further concentration and characterization of this elution will be conducted in future studies.

## 4. Discussion

Vulvovaginal candidiasis (VVC), commonly known to be caused by *C. albicans* and occurring in recurrent forms, is one of the most common vaginal discharges affecting women during their reproductive age [1]. It is strongly correlated with the dysbiosis of the normal vaginal flora [33]. The clinical symptoms of vulvovaginal candidiasis negatively affect women’s quality of life, often leading to a request for azole antifungal drugs, primarily clotrimazole, miconazole, and fluconazole, which can be administered topically or orally. A decrease in *Lactobacillus*-dominated microbiota, which is considered a valuable biomarker for vaginal health, may facilitate the overgrowth of *Candida* population [34]. *L. crispatus*, *L. jensenii*, and *L. gasseri* are the most frequently isolated species in the vagina of healthy women, which have vaginal pH levels of 4.0, 4.7, and 5.0, respectively. In these cases, *C. albicans* is often a minority cohabitant of the vaginal microbiota [35]. While conventional antifungal drugs are effective treatments for VVC, probiotic supplementations, especially Lactobacillus, have been suggested as an alternative way for women to restore vaginal health [36]. In the present study, our aim was to evaluate the anticandidal properties of lactic acid bacteria isolates, identify active lactobacilli, and assess their impact on *Candida* growth and biofilm formation, which are associated with antimicrobial resistance [37]. An effective strategy involving the use of lactobacilli and their active compounds against *Candida* infections could reduce *Candida* growth in the planktonic form as well as impair *Candida* biofilm formation *in vitro*.

Our data suggest that the tested lactic acid bacteria isolates, especially *Lactobacillus,* exhibited moderate to strong anticandidal activity. We were able to isolate potent isolates belonging to *Lacticaseibacillus rhamnosus*, *Latilactobacillus curvatus*, *Limosilactobacillus fermentum*, *Lactobacillus crispatus*, *Lacticaseibacillus paracasei* subsp. *paracasei*, and *Limosilactobacillus reuteri.* Previously, our colleagues in the laboratory reported the antimicrobial properties of lactic acid bacteria, especially *L. crispatus,* for inhibiting some pathogens [26]. Moreover, we also reported some strains of lactobacilli that inhibited the growth of *C. albicans* [19], which was in line with the study conducted by Parolin et al. [38]. The predominance of *L*. *crispatus* was strongly associated with normal vaginal microbiota and the absence of vaginal dysbiosis [39]. In the present study, a probiotic combination of *L. crispatus* and *L. reuteri* was selected for investigating the antagonistic effect on *Candida* planktonic growth and the anti-biofilm potential.

Lactic acid bacteria, especially lactobacilli, are known for producing various antimicrobial compounds to prevent vaginal infection [40]. The production of organic acids, particularly lactic acid, has been frequently correlated with the anticandidal activity of *Lactobacillus,* resulting in a lowered vaginal pH [41]. Although hydrogen peroxide and lactate are strongly associated with the antimicrobial activity of *Lactobacillus* [42], we found that our selected isolates of *L. crispatus* and *L. reuteri* had lower H_2_O_2_ production abilities compared to other lactic acid bacteria isolates. Organic acid production, particularly lactic acid, and the consequent pH reduction due to lactobacilli growth have been suggested as one of the mechanisms for inhibiting *Candida* growth in previous studies [43]. However, we found that the anticandidal activities of the tested probiotic combination may not be primarily attributed to a low pH resulting from organic acid production. Presently, the fungicidal activities were evaluated against *C*. *albicans* and *C*. non-*albicans*. Compared to previous studies focusing on the antifungal activity of lactobacilli [44], our work provided the additional value of examining *Lactobacillus* isolates against a broad spectrum of *Candida* species, including the most represented species responsible for VVC.

Here, we demonstrated that inhibitory activity against *Candida* and biofilm formation was higher in a synbiotic culture of the probiotic combination than in *L. crispatus* or *L. reuteri* isolates alone. This suggests that other antimicrobial compounds may also contribute to the *C. albicans* growth inhibition. We, thus, speculated that anticandidal activity may be involved, such as the release of bacteriocins, which can prevent *Candida* biofilm formation. The probiotic combination of *L*. *crispatus*, and *L*. *reuteri* exhibited the broadest spectrum of activity, demonstrating fungicidal activity against all isolates of both *C*. *albicans* and non-*C*. *albicans*. Among these strains, this combination could be symbiotically cultured with plant powder extract and exhibited an enhancement of anticandidal activity. In particular, the probiotic combination of *L*. *crispatus* 84/7 and *L. reuteri* 89/4 supplemented with *H. tuberosus* powder extract appeared to be the most effective in reducing pathogen growth and biofilm formation, as their effects were mediated by the supernatant. The collected cell-free supernatant will be investigated to identify the mechanism of anticandidal activity, including exclusion, competition, displacement, and killing, as previously reported [45]. In addition to lactic acid and H_2_O_2_ production, as described above, analyzing the antimicrobial activity of the selected synbiotic culture’s cell-free supernatant is crucial for identifying specific bioactive compounds and understanding the anticandidal mechanisms of vaginal lactobacilli.

Bacteriocins have been suggested to contribute to the antagonistic effects of probiotic *Lactobacillus* strains against a variety of vaginal pathogens [46,47]. The heat-stable property of bacteriocin reported in this study is consistent with other reports about the heat stability of bacteriocins [15,48,49]. The anticandidal activity of bacteriocin could be detected under acidic, neutral, and alkaline conditions (with stable activity in a pH range of 2 to 8), indicating that this activity is not a result of acid production. The inactivation of bacteriocin by proteolytic enzymes was detected, suggesting its proteinaceous nature [50]. Finally, the anticandidal activity of bacteriocin was not due to hydrogen peroxide, as its activity was maintained after treatment with catalase. After purification using anionic exchange chromatography, we were able to detect the anticandidal portion in the second elution of crude protein harvested from the cell-free supernatant of a synbiotic culture involving two selected mixed isolates with *H. tuberosus* powder extract. It also revealed two distinct protein bands. These bands may be attributed to *L. crispatus* 84/7, as reported in our previous study [26]. These bands cannot be isolated using gel filtration to be differentiated by molecular weight. However, further proteomic characterization of these bands and determination of their amino acid sequences will be conducted.

These findings suggest that supplementing the probiotic combination with selected plant powder extract enhances its anticandidal activity, especially when grown in biofilm form. This confirms the desirability of inhibiting biofilms with this synbiotic culture in the vaginal niche, aiming to not only impede candida growth but also hinder functional biofilm formation. Although obtained through in vitro tests, these findings confirm the potential of the probiotic combination of *L. crispatus* and *L. reuteri* strains, which exhibits the strongest anticandidal activity and emphasizes the significance of detecting the vaginal *L. crispatus* in the vaginal microbiome as a hallmark. Hence, they should undergo further characterization for safety in molecular and cellular models, as well as in an in vivo study, with the aim of potentially utilizing these probiotic strains to prevent VVC. In light of possible applications, these results suggest that this probiotic combination with plant powder extract formula is a strong candidate for the development of a synbiotic product, enhancing its therapeutic properties.

## 5. Conclusions

It has been concluded that synbiotic cultures formed by combining two selected anticandidal isolates, *L. crispatus* and *L. reuteri*, with a potent plant powder extract from *H. tuberosus* can produce bacteriocin with inhibitory activity against the growth and biofilm formation of *C. albicans*. It is suggested that supplementing this probiotic combination could lead to the production of anticandidal bacteriocins, enhancing growth inhibition and biofilm disruption. Further proteomic characterization, purification, and large-scale production will be conducted for therapeutic applications.

## Figures and Tables

**Table 1:** LAB species recruited in the present study. Some species of LAB were recently classified.

LAB Species	Numbers of Isolates
*Bifidobacteria*, *Lactobacilli*, *Lactococci*, and *Pediococci*:	
*Bifidobacterium lactis*	6
*Bifidobacterium longum*	12
*Lactobacillus acidophilus*	27
*Lactobacillus crispatus*	27
*Lactobacillus johnsonii*	15
*Lactobacillus delbrueckii* subsp. *delbrueckii*	33
*Lactobacillus acetotolerans*	4
*Lactobacillus helveticus*	21
*Lactobacillus jensenii*	9
*Lactococcus lactis*	33
*Pediococcus acidilactici*	36
Homofermentative LABs:	
*Companilactobacillus kimchi* *	1
*Fructilactobacillus fructivorans* *	1
*Lacticaseibacillus casei* *	24
*Lacticaseibacillus paracasei* subsp. *paracasei* *	90
*Lacticaseibacillus rhamnosus* *	27
*Latilactobacillus sakei* subsp. *sakei* *	15
*Latilactobacillus curvatus* *	21
*Lactiplantibacillus plantarum* subsp. *plantarum* *	90
*Lentilactobacillus buchneri* *	5
*Lentilactobacillus kefiri* *	1
*Levilactobacillus brevis* *	3
*Levilactobacillus cerevisiae* *	1
*Liquorilactobacillus capillatus* *	3
Heterofermentative LABs:	
*Limosilactobacillus fermentum* *	54
*Limosilactobacillus reuteri* *	39
*Secundilactobacillus kimchicus* *	1
*Secundilactobacillus oryzae* *	1

* The basonym of each species was described in our previous study [26].

**Table 2:** Lists of the tested cereals, vegetables, tuber plants, and fruits.

No.	Scientific Names	Thai Names	General Names
Cereals			
1	*Bruguiera cylindrica*	Ma-led-tua-kao	Navy bean
2	*Cajanus cajan*	Tua-rae	Pigeon pea
3	*Glycine max*	Tua-lueng	Soybean
4	*Phaseolus vulgaris*	Tua-daeng	Red bean
5	*Sesamum indicum*	Nga-dum	Black sesame
6	*Zea mays*	Kao-pod	Sweet corn
Vegetables			
7	*Brassica oleracea*	Broc-co-li	Broccoli
8	*Cucurbita moschata*	Fag-thong	Pumpkin
9	*Cynara scolymus*	Ar-ti-choke	Globe artichoke
10	*Sechium edule*	Cha-yo-te	Chayote
11	*Solanum melongena* var. *serpentinum*	Ma-kue-maung	Eggplant
Fruits			
12	*Ananas comosus*	Sup-pa-rod	Pineapple
13	*Averrhoa carambola*	Ma-fueng	Star fruit
14	*Citrus maxima*	Som-o-thong-dee	Pummelo
15	*Hylocereus undatus*	Kaew-mung-korn	Dragon fruit
16	*Malus domestica*	Ap-ple	Gala apple
17	*Passiflora edulis*	Saw-wa-rod	Passion fruit
18	*Prunus salicina*	Luk-nai-daeng	Japanese plum
19	*Pyrus pyrifolia*	Sa-lee-hi-mah	Nashi pear
20	*Vitis labrusca*	A-ngoon-moung	Purple table grapes
Medicinal plants			
21	*Angelica sinensis*	Tung-kui	Dong quai
22	*Kaempferia parviflora*	Kra-chai-dum	Black ginger
Tuber plants			
23	*Arctium lappa*	Som-jak-ka-pat	Greater burdock
24	*Daucus carota*	Car-rot	Carrot
25	*Helianthus tuberosus*	Kaen-ta-wan	Jerusalem artichoke
26	*Raphanus sativus* var. *longipinnatus*	Hua-chi-tao	Chinese radish

**Table 3:** The inhibition zones of 41 selected LABs after they were determined by agar-well diffusion.

LAB Species	*S. aureus* ATCC 25923	*S. lutea* ATCC 9341	*E. coli* ATCC 29213	*B. subtilis* ATCC 6633
*L. acetotolerans* 601	24.00 ± 0.50 *	20.00 ± 1.00	20.00 ± 0.87	22.50 ± 1.50
*L. acidophilus* 18/1	22.50 ± 0.67 *	22.00 ± 1.67	18.50 ± 0.33	21.50 ± 1.25
*L. buchneri* D09	22.00 ± 0.50	21.50 ± 0.50	24.00 ± 0.50 *	28.50 ± 0.67 *
*L. buchneri* D33	22.00 ± 1.50	25.50 ± 0.87 *	22.50 ± 0.67	25.50 ± 1.50 *
*L. crispatus* 23/1	20.00 ± 0.87	18.50 ± 0.67	22.00 ± 0.50	20.00 ± 1.50
*L. crispatus* 27/9	22.00 ± 0.50	25.50 ± 0.87 *	20.50 ± 0.76	28.50 ± 0.67 *
*L. crispatus* 33/9	22.00+1.67	21.50 ± 1.25	20.00 ± 1.00	24.00 ± 0.29 *
***L. crispatus* 84/7 #**	25.00 ± 0.87 *	31.50 ± 0.29 *	24.50 ± 0.29 *	29.50 ± 0.76 *
*L. crispatus* 55/9	18.50 ± 0.67	21.00 ± 0.87	23.50 ± 1.25 *	28.50 ± 0.67 *
*L. crispatus* 22/2	19.50 ± 0.29	17.50 ± 0.29	25.50 ± 0.87 *	20.50 ± 0.76
*L. curvatus 74/6*	22.50 ± 0.67 *	19.00 ± 0.29	18.50 ± 0.67	21.00 ± 0.29
***L. curvatus* 87/6 #**	25.00 ± 0.29 *	30.50 ± 0.87 *	20.50 ± 0.76	28.00 ± 0.87 *
*L. curvatus* 92/6	25.50 ± 0.67 *	21.50 ± 0.50	25.50 ± 0.87 *	19.50 ± 0.29
*L. delbrueckii 77/2*	22.50 ± 0.67 *	22.00 ± 0.29	18.50 ± 0.67	22.00 ± 1.67
**L. fermentum 20/6 #**	26.00 ± 1.00 *	29.50 ± 0.76 *	22.00 ± 1.00	29.50 ± 1.00 *
*L. fermentum* 32/6	23.50 ± 1.25 *	19.00 ± 0.29	18.50 ± 0.67	18.50 ± 0.33
*L. fermentum* 44/6	22.50 ± 0.67 *	23.50 ± 0.67	24.00 ± 0.50 *	22.00 ± 1.67
*L. fermentum* 48/6	19.50 ± 0.29	22.50 ± 0.67	21.50 ± 0.50	22.50 ± 0.67
*L. jensenii* 881	23.00 ± 0.50 *	19.00 ± 0.29	21.00 ± 0.87	20.00 ± 1.50
*L. lactis* H57	22.50 ± 0.67 *	22.00 ± 0.50	24.00 ± 0.29 *	22.00 ± 0.29
*L. paracasei* 6/5	22.00 ± 0.29	20.00 ± 1.00	21.50 ± 0.50	22.50 ± 1.50
***L. paracasei* 9/5 #**	25.00 ± 0.76 *	32.00 ± 0.76 *	19.50 ± 0.29	29.00 ± 0.87 *
*L. paracasei* 18/5	18.50 ± 0.67	22.00 ± 0.50	22.00 ± 1.67	19.00 ± 0.29
*L. paracasei* 41/5	21.50 ± 0.50	22.50 ± 0.67	21.00 ± 0.29	22.50 ± 0.67
*L. plantarum* 8/8	22.00 ± 0.67	22.50 ± 1.50	24.00 ± 0.29 *	28.50 ± 0.67 *
*L. plantarum* 56/8	21.00 ± 0.29	22.50 ± 0.67	23.00 ± 0.50 *	22.50 ± 1.50
*L. plantarum* 86/8	18.50 ± 0.67	22.50 ± 1.50	21.00 ± 2.00	20.00 ± 1.50
*L. plantarum* 90/8	25.50 ± 1.50 *	23.50 ± 1.25	19.00 ± 0.29	24.00 ± 0.29 *
*L. reuteri* 22/4	19.00 ± 0.29	20.00 ± 1.50	23.00 ± 0.50 *	23.50 ± 1.25 *
*L. reuteri* 66/4	21.00 ± 2.00	22.50 ± 1.50	25.50 ± 0.87 *	29.00 ± 0.29 *
*L. reuteri* 71/4	18.50 ± 0.67	22.50 ± 0.67	22.00 ± 0.50	22.00 ± 0.29
***L. reuteri* 89/4 #**	26.00 ± 0.76 *	31.50 ± 0.87 *	27.00 ± 0.29 *	29.50 ± 0.76 *
*L. reuteri B16*	21.50 ± 1.25	21.50 ± 0.50	21.00 ± 2.00	20.00 ± 1.50
*L. rhamnosus* 22/5	19.00 ± 0.29	22.00 ± 0.29	25.50 ± 0.87 *	23.50 ± 1.25 *
*L. rhamnosus* 61/7	23.50 ± 1.25 *	21.00 ± 0.29	19.50 ± 0.29	20.00 ± 1.50
***L. rhamnosus* 68/7 #**	24.50 ± 0.76 *	31.00 ± 0.29 *	21.00 ± 0.76	23.50 ± 0.76 *
*L. rhamnosus* 83/7	20.00 ± 1.00	18.50 ± 0.33	22.50 ± 0.67	24.00 ± 0.29 *
*L. sakei* 26/7	22.50 ± 1.50 *	21.50 ± 0.50	22.00 ± 0.29	25.50 ± 0.87 *
*L. sakei* 29/7	23.00 ± 0.50 *	20.00 ± 1.50	23.50 ± 1.25 *	22.00 ± 0.50
*P. acidilactici* G02	18.50 ± 0.67	25.50 ± 0.87 *	18.50 ± 0.33	20.00 ± 0.87
*P. acidilactici* G53	21.50 ± 1.25	19.50 ± 0.29	22.00 ± 1.67	20.00 ± 1.50

Bold number, the top three largest diameters of inhibition zone of each tested isolate. *, the significantly different group of isolates that were classified by cut-point at 95% CI using Mann–Whitney test. #, the selected isolates that exhibited significantly different ≥ 3 tested indicators.

**Table 4:** Synbiotic growth of each selected isolate with plant powder extracts. Data are shown as the average fold change in bacterial growth after being compared with LM.

No.	Plants	Average Fold Change in LPM Growth after Compared with Each LM					
		LRH68/7	LCV87/6	LF20/6	LCP84/7	LPR9/5	LRE89/4
LM		1.00	1.00	1.00	1.00	1.00	1.00
1	*B. cylindrica*	1.12 ± 0.04	1.13 ± 0.13	1.10 ± 0.12	1.10 ± 0.04	1.08 ± 0.01	1.13 ± 0.03
2	*C.cajan*	1.08 ± 0.04	1.12 ± 0.01	1.09 ± 0.04	1.17 ± 0.06	1.07 ± 0.04	1.14 ± 0.02
3	*G.max*	1.12 ± 0.04	1.09 ± 0.01	1.19 ± 0.04	1.10 ± 0.01	1.17 ± 0.13	1.17 ± 0.07
4	*P. vulgaris*	1.10 ± 0.03	1.09 ± 0.07	1.09 ± 0.11	1.17 ± 0.06	1.19 ± 0.08	1.10 ± 0.03
5	*S. indicum*	1.11 ± 0.04	1.20 ± 0.16	1.19 ± 0.04	1.11 ± 0.04	1.18 ± 0.09	1.11 ± 0.02
6	***Z. mays***	1.31 ± 0.11 *	1.39 ± 0.19 *	1.32 ± 0.06 *	1.41 ± 0.06 *	1.45 ± 0.04 *	1.39 ± 0.03 *
7	*B. oleracea*	1.10 ± 0.04	1.10 ± 0.04	1.12 ± 0.01	1.19 ± 0.11	1.14 ± 0.11	1.12 ± 0.01
8	*C. moschata*	1.10 ± 0.02	1.16 ± 0.02	1.13 ± 0.06	1.15 ± 0.09	1.19 ± 0.11	1.16 ± 0.09
9	*C. scolymus*	1.12 ± 0.05	1.24 ± 0.11	1.21 ± 0.01	1.16 ± 0.07	1.16 ± 0.14	1.17 ± 0.07
10	*S. edule*	1.16 ± 0.08	1.08 ± 0.01	1.12 ± 0.01	1.12 ± 0.01	1.07 ± 0.01	1.14 ± 0.02
11	***S. melongena***	1.27 ± 0.06 *	1.25 ± 0.02 *	1.23 ± 0.06 *	1.24 ± 0.05 *	1.18 ± 0.08 *	1.22 ± 0.01 *
12	*A. comosus*	1.10 ± 0.01	1.20 ± 0.04	1.14 ± 0.08	1.13 ± 0.04	1.12 ± 0.04	1.16 ± 0.01
13	*A. carambola*	1.19 ± 0.04	1.20 ± 0.05	1.13 ± 0.01	1.13 ± 0.07	1.12 ± 0.06	1.16 ± 0.02
14	*C. maxima*	1.19 ± 0.11	1.17 ± 0.01	1.09 ± 0.01	1.16 ± 0.01	1.13 ± 0.06	1.10 ± 0.08
15	*H. undatus*	1.23 ± 0.17	1.16 ± 0.10	1.11 ± 0.01	1.10 ± 0.01	1.17 ± 0.05	1.14 ± 0.06
16	*M. domestica*	1.20 ± 0.11	1.11 ± 0.03	1.12 ± 0.03	1.16 ± 0.18	1.13 ± 0.02	1.16 ± 0.01
17	*P. edulis*	1.14 ± 0.01	1.11 ± 0.01	1.09 ± 0.01	1.18 ± 0.06	1.14 ± 0.02	1.22 ± 0.08
18	*P. salicina*	1.09 ± 0.03	1.09 ± 0.04	1.13 ± 0.01	1.22 ± 0.09	1.10 ± 0.03	1.11 ± 0.16
19	***P. pyrifolia***	1.42 ± 0.05 *	1.44 ± 0.06 *	1.44 ± 0.09 *	1.46 ± 0.07 *	1.42 ± 0.04 *	1.38 ± 0.01 *
20	*V. labrusca*	1.25 ± 0.06	1.14 ± 0.04	1.11 ± 0.01	1.14 ± 0.01	1.16 ± 0.01	1.22 ± 0.01
21	*A. sinensis*	1.07 ± 0.08	1.13 ± 0.02	1.08 ± 0.10	1.18 ± 0.17	1.17 ± 0.10	1.23 ± 0.01
22	*K. parviflora*	1.06 ± 0.11	1.07 ± 0.06	1.13 ± 0.04	1.16 ± 0.11	1.16 ± 0.03	1.17 ± 0.08
23	*A. lappa*	1.17 ± 0.05	1.15 ± 0.06	1.15 ± 0.09	1.10 ± 0.07	1.15 ± 0.04	1.20 ± 0.01
24	*D. carota*	1.11 ± 0.03	1.16 ± 0.09	1.16 ± 0.05	1.14 ± 0.11	1.11 ± 0.04	1.17 ± 0.01
25	***H. tuberosus***	1.42 ± 0.08 *	1.41 ± 0.04 *	1.46 ± 0.05 *	1.44 ± 0.11 *	1.41 ± 0.11 *	1.43 ± 0.10 *
26	*R. sativus*	1.07 ± 0.02	1.11 ± 0.02	1.11 ± 0.03	1.18 ± 0.04	1.13 ± 0.06	1.18 ± 0.01

LRH68/7, *L. rhamnosus* 68/7; LCP84/7, *L. crispatus* 84/7; LCV87/6, *L. curvatus* 87/6; LF20/6, *L. fermentum* 20/6; LPR9/5, *L. paracasei* subsp. *paracasei* 9/5; LRE89/4, *L. reuteri* 89/4. * indicates the plant species that present the biggest fold change in LPM growth.

**Table 5:** Anticandidal effect of 24-h synbiotic culture (LPM) compared with probiotic culture alone (LM).

Probiotics	Average Diameter of Inhibition Zone (mm) against *C. albicans* ATCC10231 of		LPM/LM
	LPM	LM	
LCP84/7 plus LRE 89/4	33.33 ± 0.58	24.17 ± 0.29	1.38
LCP84/7 alone	28.33 ± 0.58	23.33 ± 0.58	1.21
LRE 89/4 alone	27.67 ± 0.58	22.67 ± 0.58	1.22

*H. tuberosus* powder suspension (PM) showed only 8.67 ± 0.58 mm of average diameter of inhibition zone.

## Data Availability

Data are contained within the article.
